# Three-Dimensional Printing in Hand Surgery: What Is New? A Systematic Review

**DOI:** 10.3390/jpm15120611

**Published:** 2025-12-08

**Authors:** Said Dababneh, Nadine Dababneh, Omar El Sewify, Jack Legler, Xiya Ma, Chung Ming Chan, Alain Danino, Johnny I. Efanov

**Affiliations:** 1 Faculty of Medecine, University of Montreal, Montreal, QC H2X 3E4, Canada; 2 Faculty of Medicine and Health Sciences, Laval University, Quebec, QC G1V 0A6, Canada; 3 Faculty of Medicine and Health Sciences, McGill University, Montreal, QC H2X 3E4, Canada; 4 Plastic and Reconstructive Surgery, Department of Surgery, University of Montreal, Montreal, QC H2X 3E4, Canada; 5 Hand and Reconstructive Microsurgery, National University Hospital (NUH), Singapore 119228, Singapore; 6 Plastic and Reconstructive Surgery, Department of Surgery, Centre Hospitalier de l’Université de Montréal (CHUM), 1051 Rue Sanguinet, Montreal, QC H2X 0C1, Canada

**Keywords:** three-dimensional, printing, hand, surgery, systematic review

## Abstract

**Aim:** Preoperative planning and in-office patient education are essential elements of clinical management in patients afflicted with hand injuries. Three-dimensional (3D) printing aims to tackle these challenges by converting feedstock material into solid replicas. The purpose of this study was to review the clinical uses for 3D printing in hand surgery to identify approaches for delivering more personalized treatment strategies. **Method:** A systematic review was completed following PRISMA guidelines using Medline, Embase, and CINAHL databases, identifying studies published between 2013 and January 2025. A two-stage screening process, involving title, abstract, and full text reviews, was performed independently by two reviewers. Eligible studies included those involving patients with hand or wrist injuries (up to the distal radius) where 3D printing was utilized for diagnosis, surgical intervention, or rehabilitation. **Results:** The review included 751 patients (mean age: 38 years, range: 5–81 years) across 58 studies. The distal radius was the most commonly studied anatomical region (47%, N = 27), followed by the scaphoid (19%, N = 11). Key applications of 3D printing included preoperative planning (19%, N = 11), patient education (5%, N = 3), medical training (7%, N = 4), intra-operative assistance (38%, N = 22), splinting and casting (19%, N = 11), and prothesis and functional reconstruction (12%, N = 7). **Conclusions:** Despite its early stage of adoption in hand surgery, 3D printing has shown advantages, especially in enabling more personalized treatment strategies by improving intra-operative assistance, preoperative planning, and patient education. Further research is required to determine whether it positively affects postoperative outcomes, to calculate the cost–benefit ratio, and to compare its usage against standards of care.

## 1. Introduction

Successful management of hand and wrist injuries relies on thorough preoperative planning, patient education, and precise surgical execution [1,2]. While advancements in imaging techniques and digital learning tools have improved diagnostic accuracy and treatment strategies, certain complex injuries remain difficult to address. Three-dimensional (3D) printing, often referred to as “rapid prototyping”, has emerged as a transformative technology, allowing surgeons to create tangible anatomical models from digital imaging data. By converting raw materials into physical replicas, 3D printing enhances both patient understanding and surgical preparation.

Once a field primarily managed by biomedical engineers, 3D printing is now becoming increasingly accessible to surgeons. This shift has enabled a more personalized and informed approach to patient care [3,4,5]. The process begins with high-resolution imaging techniques, such as Computed Tomography (CT scan) or Magnetic Resonance Imaging (MRI), which capture the injury in fine detail. These images are then processed into a digital file, allowing a 3D printer to generate an exact model of the patient’s anatomy. Such models can guide preoperative decision-making, optimize the placement of fixation devices such as plates and screws, and provide real-time assistance during surgery [6,7,8,9]. With a remarkable precision of up to 0.1 mm [10], commercially available 3D printers can produce highly detailed anatomical structures, reducing surgical uncertainty and lowering the risk of complications, ultimately improving patient outcomes [11].

The applications of 3D printing extend across multiple surgical specialties. In maxillofacial surgery, for example, patient-specific bone substitutes have been successfully created, while in orthopedics, 3D models facilitate preoperative simulations for complex fractures [12,13,14,15]. Within plastic and reconstructive surgery, this technology has revolutionized procedures such rhinoplasty and peripheral nerve reconstruction [16,17]. Given its proven utility in various disciplines, it is within reason to discern the use of 3D models in reconstructive hand surgery.

Although the use of 3D printing in hand and wrist surgery is still evolving, these models hold great potential to bridge the gap between conventional two-dimensional imaging and real-world surgical fields [18]. Nevertheless, widespread adoption is limited by economic constraints, technical limitations, and the time required for model production [19,20,21].

This review aims to provide a synthesis of current literature on the role of 3D printing in hand and wrist surgery, addressing the current lack of standardized outcome assessment, cost-effectiveness data, and validation of model accuracy. Given the intricate anatomy and small operative field of the hand and wrist, this subspecialty may particularly benefit from 3D printing’s ability to support personalized treatment strategies by enhancing spatial understanding and improving precision in reconstructive procedures.

## 2. Methods

This systematic review was conducted in accordance with the Preferred Reporting Items for Systematic Reviews and Meta-Analyses (PRISMA) guidelines (Supplementary Materials) [22]. The study protocol is registered on Open Science Framework (https://doi.org/10.17605/OSF.IO/PWZ89).

### 2.1. Search Strategy

A comprehensive literature search was conducted across Medline, Embase, and CINAHL databases, initially without restrictions on language or publication type, to ensure a broad scope of retrieval. The search strategy incorporated the terms; “3D printing”, three-dimensional printing”, “additive manufacturing”, “rapid prototyping” and “hand”, “wrist”, “carpal”, “scaphoid”, “lunate”, “distal radius”, “ulna”. Boolean operators “AND” and “OR” were utilized to optimize the search.

All retrieved citations were imported into EndNote^TM^ X9, where duplicates were removed. Additional relevant studies were identified through manual reference list screening of included articles. Recognizing the rapidly evolving nature of this field, a follow-up search was conducted on 2 January 2025 to ensure the inclusion of the most up-to-date literature. This second search applied refined filters, limiting results to articles published from 2021 onwards, written in English, and available as full-text studies.

### 2.2. Study Selection

References retrieved from the initial search were imported into Rayyan, while those from the second search were managed using Covidence, both widely utilized online review platforms. Using two platforms allowed for optimized workflow management and ensured inclusion of recent publications. Data integrity across both tools was maintained by performing a final deduplication and reconciliation step before data extraction. On both occasions, title and abstract screening was conducted by two independent reviewers, followed by a detailed full-text review. Inter-rater reliability between the two independent reviewers was assessed using Cohen’s kappa coefficient to ensure consistency in study selection. At this stage, studies published between 2013 and January 2025 that involved patients with hand or wrist injuries (up to the distal radius) and the use of 3D printing for diagnosis, surgical intervention, or rehabilitation were included. Studies involving non-human models were excluded. The detailed inclusion and exclusion criteria are outlined in Table 1. Discrepancies were resolved through discussion and consensus with a third reviewer.

### 2.3. Study Eligibility

Eligible publication types included clinical trials (phases I–IV), case reports, classical articles, comparative studies, observational studies, randomized controlled trials, and validation studies. In contrast, systematic reviews, meta-analyses, letters to the editor, viewpoints, commentaries, and abstracts without full text were excluded. There were no age restrictions, allowing the inclusion of both adults (≥18 years old) and children (<18 years old). All patients with hand or wrist injuries extending up to the distal radius were eligible for inclusion, regardless of injury etiology.

## 3. Results

**Figure 1:**
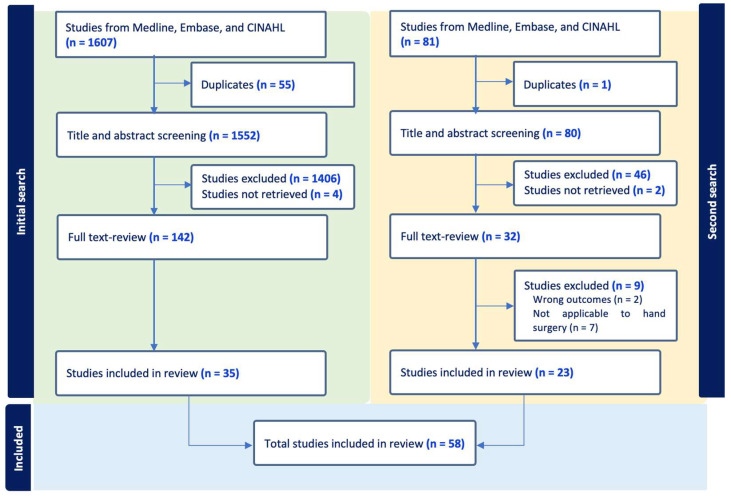
Flowchart detailing the systematic review process.

The initial search identified a total of 1607 articles. After removing 55 duplicates, 1552 citations underwent title and abstract screening. Of these, 1406 excluded articles weren’t aligned with the inclusion criteria, 4 could not be retrieved, leaving 142 articles eligible for full-text assessment. Ultimately, 35 articles met the inclusion criteria for this systematic review. The second search identified 81 articles, of which 23 met the inclusion criteria after screening and full-text review. Combined, a total of 58 studies were included in the final analysis, shown in Figure 1.

### 3.1. Study Characteristics

The studies included in this review were published between January 2013 and January 2025 and originated primarily in China (20.7%, N = 12 studies), as detailed in Table 2. The most common study design was case reports and case series, representing 60.3% (N = 22 studies), while 15 studies were comparative in nature. The distal radius emerged as the most frequently studied anatomical region, featured in 46.6% (N = 27 studies) of the publications. Other notable anatomical areas included the scaphoid (N = 11 studies), 5th metacarpal bone (N = 3 studies), and the lunate (N = 3 studies).

### 3.2. Patient Characteristics

Across 58 studies, 751 patients were included in this analysis. The mean patient age was 38 years (SD: 14, range: 5–81 years). Gender distribution (reported in 50 studies, N = 602) was balanced, (female: 49.8%, male: 50.2%). A total of 21 distinct diagnoses were described, with acute distal radius fracture being the most frequently reported (N = 19). Injuries affecting the dominant hand were noted in 13 studies. Finally, the time from injury to intervention, reported in 34 studies, ranged from 12 h to 5.8 years. Detailed demographics are provided in Table 3.

### 3.3. Clinical Uses

(1) Preoperative Planning

Eleven studies (19%) described the use of 3D-printed models for preoperative planning, primarily for complex or anatomically altered cases that require personalized medicine-driven approaches.

Eleven studies (19%) reported the use of 3D printing for preoperative planning, primarily for complex fractures, revision cases, or anatomically altered sites [34]. Studies demonstrated that patient-specific 3D models enabled improved visualization, optimized fixation selection, and enhanced surgical decision-making. In comparative analyses, 3D-assisted planning resulted in shorter operative times and lower complication rates (6–10%) compared with conventional planning (25–30%). Notably, in Grinčuk et al., major complications, such as mispositioned screws and loss of reduction, were only observed in the control group [34]. Moreover, high-fidelity printed models improved spatial understanding, fixation strategy, and implant selection. Costs averaged GBP 30–40 per model, with production times ranging from several hours to 6 days [52].

Therefore, across all evidence levels, 3D-printed models improved visualization and planning accuracy, with the strongest benefits observed in complex or revision settings where personalized medicine is most impactful. However, variability in production time, effort required, and cost remain practical limitations [27].

(2) Patient Education

Three studies highlighted the educational benefits of using 3D-printed anatomical models, demonstrating their potential to bridge communication gaps between surgeons and patients. These interventions consistently improved comprehension of injury patterns, procedural steps, and surgical rationale, thereby supporting personalized medicine. Survey data revealed higher patient satisfaction and confidence during informed consent [57]. Quantitative outcomes were rarely reported, but the qualitative impact on understanding and shared decision-making was substantial, leading to increased treatment compliance and greater confidence in their surgical team [36].

Thus, the use of 3D models effectively enhanced patient comprehension and engagement, though evidence remains limited to descriptive data without controlled comparisons.

(3) Medical and Surgical Training

The integration of 3D printing into medical education and surgical training is gaining momentum, as evidenced by four studies in this review. This technology enhanced fracture visualization, simulation-based learning, and preoperative planning, equipping trainees with practical, hands-on experience and supporting the principles of personalized medicine before encountering real-life surgical cases.

Training with 3D-printed models improved learner confidence, spatial reasoning, and OSATS (Objective Structured Assessment of Technical Skills) performance [54]. Participants rated anatomical realism highly, particularly for intra-articular fracture simulations, severe displacement (≥2 mm articular gaps), or multiple fragments [38]. Compared with traditional didactic or cadaveric teaching, trainees demonstrated faster skill acquisition and improved procedural planning [26].

Therefore, 3D printing enhanced procedural education and psychomotor learning, though the current evidence is based primarily on small, non-comparative studies.

(4) Intraoperative Assistance

3D printing has the potential to revolutionize intraoperative approaches in hand surgery by providing precise, patient-specific solutions, particularly for complex cases. Twenty-two studies evaluated intraoperative applications, including surgical guides, customized implants, and resection templates.

RCTs and comparative studies demonstrated consistent benefits in operative metrics: mean operative time decreased by 10–40%, and fluoroscopy use by 40–50%, compared with standard techniques [7,32,38]. Functional outcomes measured using DASH, PRWE, or VAS scores improved significantly in 11 of 15 comparative studies. In case reports, patients experienced complete pain relief, no local recurrence, and satisfactory functional recovery with the use of custom 3D-printed prosthesis [29,32].

Overall, the studies demonstrated a significant reduction in operative time and intraoperative fluoroscopy use, without an increase in surgical complications. Moreover, 3D-printed implants proved to be a viable alternative to conventional options such as tendon transfers, titanium prostheses, and silicone implants, enabling restoration of wrist mobility, grip strength, and pain relief with improved functional outcomes [56,73].

(5) Splinting and Casting

Eleven studies explored 3D-printed orthoses for postoperative immobilization and fracture management. In randomized trials, 3D-printed splints achieved equivalent radiological healing, pain management, and functional recovery compared with fiberglass casts while providing superior ventilation, lighter weight, and better hygiene [31,35,41]. Moreover, multiple studies report unanimous patients’ preference for 3D-printed cast over traditional alternatives for future use [28,31].

Beyond fracture care, Schutz et al. described the use of 3D-printed orthoses for correcting congenital hand deformities and joint contractures in neonates [60]. Their case series demonstrated cost-effective, lightweight, and patient-specific solutions for neonates.

Therefore, 3D-printed splints matched the therapeutic effectiveness of conventional casts while improving comfort and patient satisfaction, supporting a more personalized medicine approach to immobilization.

(6) Prosthetics and Functional Reconstruction

Seven studies reported prosthetic applications, including custom finger and transradial prostheses, as well as reconstructive implants for Kienböck’s disease or bone loss. These devices improved grip function and offered better comfort while also allowing for quicker production and reduced costs compared to conventional methods [64].

Therefore, patients demonstrated meaningful functional improvements in daily tasks while benefiting from the low cost, rapid production, and high degree of customization offered by 3D printing, reinforcing its role in personalized medicine. However, the authors also identified material durability and long-term wear as limitations requiring further refinement [47].

### 3.4. Postoperative Outcomes

Follow-up durations were reported in 42 studies, with a mean of 11.3 months (range: 0.24–48.5 months). The most frequently assessed outcome was radiological improvement, documented in 32 studies. Twenty-nine articles evaluated postoperative range of motion (ROM). Pain was assessed using the Visual Analogue Scale (VAS) in 19 studies. A variety of patient assessment questionnaires were used in 27 studies, including Disabilities of the Arm, Shoulder and Hand (DASH), Michigan Hand Outcome Questionnaire (MHQ), Quebec User Evaluation of Satisfaction with Assistive Technology (QUEST), Orthotics and Prosthetics User’s Survey (OPUS), Upper Extremity Functional Scale (UEFS), and Canadian Survey on Disability (CSD). Complications were observed in 20 out of 179 patients (11%) across 18 studies, including both major and minor issues. Major complications included deep infection and 3D models failures that led to implant removal. Minor complications consisted of issues such as screws that were too long, causing symptoms like tendon irritation, and temporary paraesthesia due to the prosthesis, which required adjustments.

Additional functional outcomes were also assessed in several studies, including grip strength (16 studies), Mayo wrist score (2 studies), modified Mayo score (2 studies), patient-rated wrist evaluation (PRWE) score (4 studies), patient-rated wrist/hand evaluation (PRWHE) score (1 study), and Jebsen Hand Function Test (1 study). Other assessments included maximum load (1 study), Michigan Hand Questionnaire (2 studies), Capitolunate, scapholunate angle measurement (1 study), Perdue Pegboard Test (N = 1), and Gartland–Werley score (1 study). The wide range of outcomes assessed, along with the heterogeneity of the results and small sample sizes, limited the ability to conduct pooled analysis.

The collective evidence supports functional improvement and acceptable complication rates with 3D-assisted interventions, though methodological heterogeneity limits quantitative synthesis.

### 3.5. Printer Characteristics

The type of printer and material used in the 3D models were specified in 41 studies (70.7%). The most common materials were polylactic acid (N = 13), resin (N = 9), and acrylonitrile butadiene styrene (N = 6). Manufacturing time was reported in 25 studies, ranging from 25 min to 144 h. Cost per 3D printed model was noted in 24 articles, whereas printer cost was detailed in only two studies. Further details are provided in Table 4.

## 4. Discussion

This systematic review synthesizes current evidence on 3D printing in hand and wrist surgery, encompassing preoperative planning, patient education, surgical simulation, intraoperative assistance, splinting, and prosthetics. Current evidence suggests that 3D printing enhances anatomical visualization, facilitates surgical planning, reduces operative time and fluoroscopy use, supports patient and trainee education, and enables personalized medicine through patient-specific approaches.

### 4.1. Quality of Evidence

The literature is dominated by case reports and small case series, which, while illustrative, provide limited generalizability. Only a minority of studies (26%) employed comparative or randomized designs. Among these, consistent findings included reduced operative time, fewer fluoroscopy exposures, and improved anatomical accuracy. The heterogeneity of methodologies, outcomes, and follow-up periods hindered meta-analytic synthesis. Nevertheless, the convergence of findings across multiple low- and moderate-level studies lends credibility to the observed trends.

### 4.2. Functional and Operative Benefits

Across comparative studies, 3D models offer surgeons a tangible representation of patient’s anatomy, facilitating pre-surgical simulations [74]. These models enable surgeons to refine their technique prior to surgery [28,74] and tailor surgical tools and materials to the unique anatomical characteristics of each patient [29] Ultimately, these adaptations to pre-surgical planning consistently shortened surgical duration and decreased intraoperative radiation exposure without increasing complication rates [7,19,29,36]. As highlighted in our review, studies such as that by Casari et al. have demonstrated that these advancements have been associated with improved postinterventional outcomes, including enhanced range of motion, better wrist functionality, greater accuracy in fracture reductions, and a reduced incidence of adverse events [27].

Improvements in functional outcomes, particularly DASH, VAS, and PRWE scores, were evident in over 70% of studies using validated tools. These benefits were most pronounced in complex fractures, revision surgeries, and tumor resections requiring customized implants. Simpler fractures, conversely, showed less incremental advantage over conventional methods.

### 4.3. Complications and Safety

The observed complication rate of 11% across 18 studies should be interpreted cautiously. While it includes both major and minor issues, the nature and severity of these complications were inconsistently reported. Many studies did not clearly distinguish between complications directly related to 3D printing and those arising from surgical technique or patient factors. The study by Grincuk et al., which specifically compared distal radius fracture management, did show a lower complication rate in the 3D model group; however, as a single study, it cannot offset the pooled complication rate across all studies [34]. Despite this, the available evidence suggests that 3D printing can enhance surgical planning and may help reduce certain complications, but further well-designed studies are needed to clarify its true impact on patient outcomes.

### 4.4. Cost and Feasibility

Economic feasibility remains a critical consideration for the implementation of 3D models in healthcare [19], with printing and materials expenses ranging from USD 10 to 2000 per surgery depending on complexity and material. Additionally, 3D printing may lead to long-term savings due to shorter operative times [47], which costs approximately USD 30 per minute in the operating room [36]. Furthermore, once created, these models can quickly be modified to the anatomical specifications of patients with similar pathological features, reducing the time needed for new constructions [36]. Therefore, despite the initial costs of equipment and extra time spent preoperatively, 3D-printing technology represents as a long-term investment [3]. Moreover, declining printer and material prices are likely to further enhance accessibility, especially for teaching hospitals and high-volume trauma centers [75]. Nevertheless, further cost-analysis studies are needed to evaluate the financial benefits of 3D model implementation.

### 4.5. Educational and Patient-Centered Value

Patient education and trainee instruction benefit from the tangible, visual nature of 3D models. Evidence indicates improved patient comprehension and trainee skill acquisition, particularly in complex fractures. Intraoperative applications, including patient-specific guides and implants, demonstrate reproducible reductions in operative duration and radiation exposure. Prosthetic and reconstructive uses show promise in functional recovery, though long-term durability and cost-effectiveness remain underexplored.

### 4.6. Evidence Gaps and Future Directions

This systematic review has several limitations, including the exclusion of non-English studies, potentially overlooking valuable contributions.

Despite encouraging findings, the literature lacks high-level evidence and long-term outcome data. Standardized reporting of PROMs, radiological metrics, and economic data would allow more robust cross-study comparisons. Additionally, only 26% of the included studies were comparative in nature, limiting the ability to draw robust conclusions about the superiority of 3D printing over standard care in hand and wrist surgery.

Despite their advantages, several studies have reported practical limitations of 3D-printed models in hand surgery. Challenges included difficulties with surgical approach, tool positioning, and plate placement, as well as occasional design flaws and technical limitations of the printing process [5]. Furthermore, the effectiveness of 3D models appeared to depend on fracture complexity, providing the greatest benefit in complex articular or multi-fragmentary fractures but offering limited added value in simpler cases [19,76].

Nevertheless, the significant heterogeneity in methodologies, clinical applications, and patient demographics across studies posed challenges for meaningful synthesis and further pooled analyses. This diversity underscores the versatility of 3D printing and the difficulties in consolidating findings into actionable clinical guidance. Future research should focus on large, multicenter RCTs comparing 3D-assisted versus conventional surgery, incorporating objective performance metrics (e.g., operative time, fluoroscopy count) and validated functional outcomes. Additionally, exploring biodegradable or composite materials could enhance implant durability and biocompatibility.

### 4.7. Overall Interpretation

The cumulative evidence supports 3D printing as a safe, versatile, and increasingly valuable tool in hand and wrist surgery, particularly for anatomically complex or reconstructive cases. Its benefits extend beyond technical execution to encompass patient education, surgical training, and the delivery of personalized medicine through patient-specific approaches. However, to transition from innovation to standard of care, further evidence is needed to confirm long-term effectiveness, optimize costs, and establish standardized quality control.

## 5. Conclusions

The integration of 3D printing in hand surgery represents a transformative advancement, offering patient-specific solutions that embody the principles of personalized medicine. Across domains such as preoperative planning, surgical training, prosthetics, splinting, and patient education, 3D printing enhances anatomical visualization, improves procedural accuracy, and optimizes functional outcomes. Evidence demonstrates reductions in surgical time and radiation exposure, alongside improved patient comprehension and satisfaction. However, challenges such as material durability, cost-effectiveness, and broader clinical adoption remain areas for future refinement.

## Figures and Tables

**Table 1:** Inclusion and exclusion criteria of the systematic review.

	Inclusion Criteria	Exclusion Criteria
Study design	○ Randomized controlled trials (RCTs) ○ Prospective and retrospective studies ○ Case–control studies ○ Case reports and case series	○ Systematic reviews ○ Meta-analyses ○ Letters to the editor ○ Viewpoints or commentaries ○ Conference abstracts without full-text availability
Population	○ Patients with hand or wrist injuries, extending to the distal radius ○ Both pediatric (<18 years) and adult (≥18 years) populations	○ Studies involving non-human models (e.g., cadavers, animal studies) ○ Patients with injuries beyond the distal radius
Intervention	○ Use of 3D printing technology for diagnosis, preoperative planning, surgical intervention, or rehabilitation	
Outcome Measures	○ Clinical outcomes (e.g., surgical precision, functional recovery) ○ Postoperative outcomes (e.g., healing time, complication rates) ○ Patient-specific outcomes (e.g., satisfaction, quality of life)	
Publication details	○ Full-text articles available in English ○ Studies published between 2013 and 2025	

**Table 2:** Study characteristics.

Study	Year	Methodology	Comparative Study	Funding	Country	Purpose
Anderson et al. [23]	2021	Case report	No	No	USA	To investigate the use of a 3D-printed prosthetic hand in enhancing a child’s participation, confidence, and satisfaction in gymnastic classes
Belloti et al. [24]	2021	Prospective cohort	No	Yes	Brazil	Evaluate the functional and radiographic outcomes of corrective osteotomies for symptomatic distal radius malunions through planning with prototyping in 3D printing
Belloti et al. [25]	2021	Case series	No	Yes	Brazil	Prototyping based on 3D reconstruction of computed tomography scans showing its use in clinical cases
Bizzotto et al. [13]	2015	Prospective cohort	No	No	Italy	Surgeons’ appreciation of articular fragments dislocation, preoperative planning for placement fixation and patient/resident/student education
Bizzotto et al. [3]	2016	Technical note	No	Yes	Italy	Preoperative planning for placement of fixation and patient education
Brichacek et al. [26]	2018	Prospective cohort	No	No	Canada	Surgical education for K-wire placement in common hand fractures
Casari et al. [27]	2021	Prospective case series	Yes	Yes	Switzerland	Evaluate feasibility of computer-assisted surgical planning and 3D-printed patient-specific instrumentation for treatment of distal intraarticular radius fractures
Chen et al. [11]	2018	Case report	No	Yes	China	Report the use of a 3D-printed porous tantalum prosthesis in the limb-salvage surgery of a patient with distal radial osteosarcoma
Chen et al. [28]	2017	Randomized controlled trial	Yes	Yes	China	Evaluate the feasibility, accuracy, and effectiveness of applying 3D printing technology for preoperative planning for die-punch fractures
Chen et al. [29]	2021	Clinical trial	No	Yes	Hong Kong	Use 3D-printed casts, developed for distal radius fracture treatment, to provide the foundation for conducting additional clinical trials, and to perform clinical assessments
Chen et al. [7]	2019	Randomized controlled trial	Yes	Yes	China	Evaluate the use of 3D printing models for preoperative planning in cases of complex fracture
Copeland et al. [30]	2022	Case report	No	Yes	USA	Compare the functional outcomes and patient satisfaction of a standard clinic-fitted transradial prosthesis versus a remotely fitted 3D-printed prosthesis
El Khoury et al. [31]	2022	Randomized controlled trial	Yes	Yes	Belgium	Compare the effectiveness of 3D-printed splints with conventional removable splints in managing distal radius fractures
Exner et al. [32]	2021	Case report	No	No	Switzerland	Demonstrate the use of 3D printing technology to create a patient-specific mold for forming an antibiotic-loaded cement spacer in the treatment of an infected sarcomatous radius
Eyiis et al. [33]	2023	Randomized controlled crossover trial	Yes	No	Netherlands	Compare the effectiveness, patient satisfaction, and compliance of a 3D-printed customized brace versus a conventional plaster brace for the non-operative management of trapeziometacarpal osteoarthritis
Grincuk et al. [34]	2023	Prospective randomized control study	Yes	No	Lithuania	Evaluate whether the use of 3D-printed models for preoperative planning improves short-term functional outcomes and reduces complication rates in the surgical treatment of distal radius fractures
Guebeli et al. [35]	2024	Randomized controlled trial	Yes	Yes	Netherlands	Compare clinical outcomes between 3D-printed splints made of photopolymer resin and traditional fiberglass casts for the treatment of distal radial fractures
Honigmann et al. [36]	2016	Case report	No	No	Switzerland	Describe a technique using a 3D-printed drill guide and template to simplify the surgical reconstruction of a malunited distal radius fracture
Houdek et al. [37]	2016	Case report	No	No	USA	Describe a novel technique for preoperative surgical planning for an osteoarticular medial femoral condyle graft to replace the proximal pole of a scaphoid
Huang et al. [38]	2021	Case series	No	Yes	Taiwan	Demonstrate the effectiveness of a 3D-printed orthosis for improving the range of motion in patients with wrist and thumb contractures. Describe and demonstrate a newly designed device to ease splint construction, a 3D-printed “shark fin”-shaped device that works as a static progressive orthosis for hand rehabilitation
Inge et al. [19]	2018	Case report	No	No	Netherlands	3D virtual surgical planning for osteotomy and saw guides for osteotomy and iliac crest bone graft
Jew et al. [39]	2019	Case series	No	No	USA	Improve preoperative planning for a series of complex scaphoid fractures, particularly with implant choice and positioning
Joo et al. [40]	2021	Case report	No	Yes	South Korea	Evaluate the effectiveness of a 3D-printed exoskeleton robot in restoring hand function for a patient with hand dysfunction following burn injuries
Kim et al. [41]	2018	Randomized controlled trial	Yes	Yes	South Korea	Develop a personalized wrist orthosis using a 3D scanner/printer for patients with wrist pain
Kohlhauser et al. [42]	2023	Case report	No	No	Austria	Demonstrate the successful surgical management of a complex hand trauma using individualized 3D printing technology for joint reconstruction
Kong et al. [43]	2020	Randomized controlled trial	Yes	Yes	China	Evaluate the effectiveness and safety of surgical treatment of intra-articular distal radius fractures with the assistance of 3D printing technique
Krishna et al. [44]	2022	Case report	No	No	India	Demonstrate the use of 3D printing technology in creating bone models and surgical guides for minimally invasive resection of osteoid osteoma in the distal radius without intraoperative imaging
Kunz et al. [45]	2013	Case series	No	Yes	Canada	Present a method in which patient-specific instrument guides are used to navigate the alignment of the distal and proximal fragments with respect to a preoperative plan and to assist with plate fixation to achieve planned realignment
Kuptniratsaikul et al. [46]	2021	Case report	No	Yes	Thailand	Presentation of a reconstruction technique using a custom 3D-printed endoprosthesis with multiple ligament reconstruction for anatomical restoration of a large defect
Lee et al. [47]	2022	Case series	No	Yes	South Korea	Demonstrate the use of body-powered 3D-printed finger prostheses, highlighting their potential as a functional and cost-effective alternative
Ma et al. [48]	2020	Retrospective case series	No	Yes	China	Evaluate the feasibility of arthroplasty with varisized 3D printing lunate prosthesis for the treatment of advanced Kienböck’s disease
Marcano-Fernandez et al. [49]	2021	Prospective cohort	Yes	No	Spain	Compare the accuracy and reliability of percutaneous fixation of minimally displaced scaphoid fractures using a custom 3D-printed guide with a conventional freehand method
Matter-Parrat and Liverneaux [5]	2019	Case report	No	No	France	Provide how 3D printed objects can be used in radial osteotomy and its application on a clinical case
Oka et al. [50]	2020	Retrospective case series	No	Yes	Japan	Investigate five patients with malunited intra-articular distal radius fracture who underwent corrective osteotomy performed using a patient-matched instrument designed/manufactured based on preoperative 3D computer simulation
Oki et al. [51]	2021	Case report	No	Yes	Japan	Demonstrate the use of a 3D-printed model volar plate for preoperative planning in a case of scaphoid fracture non-union
Osagie et al. [52]	2017	Case series	No	No	UK	Use of 3D printing in operative planning of wrist surgery revision
Peeters et al. [53]	2020	Letter to the editor	No	No	Belgium	Use of 3D printing technology to aid in the reconstruction of scaphoid non-union humpback deformities
Raeker-Jordan et al. [54]	2022	Case report	No	No	USA	Develop and validate novel, high-fidelity 3D-printed surgical training phantoms for distal radius fracture reductions as an alternative to traditional cadaveric models
Roner et al. [55]	2018	Retrospective cohort	Yes	Yes	Switzerland	Compare the accuracy of navigation of 3D planned opening-wedge osteotomies using a ramp-guide over guide techniques relying solely on pre-drilled holes
Rossello [56]	2021	Case report	No	No	Italy	Evaluate the use of a custom-made 3D-printed titanium implant for scaphoid replacement and scapholunate ligament reconstruction in a patient with non-union and necrosis.
Samaila et al. [57]	2020	Prospective cohort	No	None	Italy	Education of patients during informed consent and models used as templates for open reduction internal fixation planification
Schmidt et al. [58]	2020	Case report	No	No	Austria	Demonstrate the use of 3D printing-assisted medial femoral condyle flap for metacarpal reconstruction following the resection of a large giant cell tumor recurrence
Schmidt et al. [59]	2022	Case series	No	None	Austria	3D virtual surgical planning for resection of proximal pole of scaphoid and intraoperative verification of medial femoral condyle flap to be harvested
Schutz et al. [60]	2022	Case series	No	No	USA	To assess the feasibility of utilizing 3D scanning, design, and printing technologies for creating customized splints for neonates
Schweizer et al. [61]	2013	Case series	No	NR	Switzerland	Analyze the feasibility of combining computer-assisted 3D planning with patient-specific drill guides and evaluate this technology’s surgical outcomes for distal radius intra-articular malunions
Sedigh et al. [62]	2022	Case series	No	No	USA	To explore the use of 3D-printed splints generated from calibrated 2D images, offering a contactless alternative to conventional splinting
Shintani et al. [63]	2018	Prospective cohort	No	NR	Japan	Investigate the results of a computer-assisted, 3D corrective osteotomy using prefabricated bone graft substitute to treat distal radius malunited fractures
Stefanovicet al. [64]	2021	Case report	No	No	Slovakia	Demonstrate the design and fabrication of an individual passive thumb prosthesis using 3D scanning and printing
Temmesfeld et al. [65]	2020	Case report	No	NR	Norward	Design and produce patient-specific surgical guides in-house with a benchtop 3D printer to perform an arthroscopy-assisted intra-articular osteotomy
Vijayan et al. [66]	2023	Case report	No	No	India	Demonstrate the use of 3D printing to create a functional prosthesis for an amputated index finger, aiming to improve functionality, efficiency, and psychological well-being
Wan et al. [67]	2019	Case series	No	Yes	China	Explore the feasibility of employing computer-aided design and 3D-printed personalized guide plate for the mini-invasive percutaneous internal screw fixation of fractured scaphoid
Wang et al. [68]	2020	Retrospective cohort	Yes	Yes	China	Compare the outcomes of an osteoarticular allograft versus a custom-made prosthesis reconstruction
Xiao et al. [69]	2024	Randomized controlled trial	Yes	Yes	China	To compare the short-term effectiveness, safety, and advantages of 3D-printed wrist casts versus polymer orthoses in the treatment of Colles fractures.
Xie et al. [70]	2018	Case report	No	Yes	China	Test the lunate prosthesis intraoperatively to stage IIIc Kienböck’s disease
Xu et al. [71]	2019	Prospective cohort	No	Yes	China	To evaluate and precisely internal fix intra-articular distal radial fracture using virtual X-ray and 3D printing technologies
Yin et al. [72]	2020	Prospective cohort	Yes	Yes	China	Determine whether a 3D-printed guiding plate system could facilitate the modified procedure for arthroscopic treatment of nondisplaced scaphoid non-union
Yuan et al. [73]	2022	Case report	No	Yes	China	To evaluate the clinical efficacy of a 3D-printed lunate prosthesis in the treatment of stage III Kienböck’s disease

3D: three-dimensional; NR: not reported.

**Table 3:** Summary of patient demographics.

N	
Patients	493 (65.6%)
Controls	258 (34.6%)
Age (years)	38 (SD: 14)
Time from injury (days)	356 [IQR: 60–652]
Sex *	
M	302 (50.2%)
F	300 (49.8%)
Anatomic Area of Interest	
Distal radius	34 (51.5%)
Metacarpals	6 (9.1%)
Phalanges	5 (7.6%)
Scaphoid	10 (15.2%)
Lunate	3 (4.5%)
Carpus	1 (1.5%)
Ulnar styloid	1 (1.5%)
Wrist	2 (3.0%)
Trapeziometacarpal joint	1 (1.5%)
Entire hand	3 (4.5%)
Diagnosis	
Distal radius fracture	20 (30.7%)
Bennett’s fracture	1 (1.5%)
Metacarpal facture	3 (4.6%)
Phalanx fracture	2 (3.1%)
Distal radius malunion/non-union	7 (10.8%)
Scaphoid avascular necrosis/malunion/non-union	8 (12.3%)
Kienböck	3 (4.6%)
Ulnar malunion/non-union	1 (1.5%)
Degenerative wrist	2 (3.1%)
Tumor	6 (9.2%)
Scaphoid fracture	2 (3.1%)
Skeletal dysplasia	1 (1.5%)
Chromosomal abnormalities	1 (1.5%)
Congenital hand deficiency	1 (1.5%)
Trapeziometacarpal osteoarthritis	1 (1.5%)
Proximal interphalangeal joint amputation	2 (3.1%)
Transradial amputation	1 (1.5%)
Burn	1 (1.5%)
Congenital absence of the thumb	1 (1.5%)
FPL rupture	1 (1.5%)

Descriptive data are presented as N (%). Parametric values are presented as mean (SD), non-parametric values are presented as median [IQR], * Sex was reported in 50 out of the 58 included studies (602/751 patients) FPL: flexor pollicis longus.

**Table 4:** Printer characteristics.

Study	Printer Used	Type of Material	Manufacture Time	Price per Surgery	Printer Cost
Anderson et al. [23]	Lulzbot TAZ 6	Polylactic Acid (PLA)	<2 days (printing, assembling)	USD 25	NR
Belloti et al. [24]	NR	Polylactic acid	NR	NR	NR
Belloti et al. [25]	3D printer (Makerbot, São Paulo, Brazil)	Polylactic acid	NR	NR	NR
Bizzotto et al. [13]	Pro-Jet 660 Color	Gypsum-dust material	4 h	USD 10/model	NR
Bizzotto et al. [3]	Stratasys uPrint SE (Stratasys Ltd. 7665 Commerce Way, Eden Prairie, MN 55344, USA)	Acrylonitrile butadiene styrene material	3–4 h	NR	NR
Brichacek et al. [26]	NR	Polyurethane foam with iron powder	NR	USD 50	NR
Casari et al. [27]	NR	NR	NR	USD 220–320/case	NR
Chen et al. [11]	3D printer (3D ORTHO; Waston Med Inc., Changzhou, Jiangsu, China)	NR	5 h (1 h pre-processing, 3–4 h printing)	NR	NR
Chen et al. [28]	Selective laser sintering (SLS) EOS P395 or Stereolithography (SLA) printer RS4500 (UnionTech, China)	Polypropylene (PP) and polyamide (PA2200)	NR	USD 150/cast	NR
Chen et al. [29]	UP BOX+ 3D printer	Polylactic Acid (PLA)	NR	NR	NR
Chen et al. [7]	3D printer (3D ORTHO; Waston Med Inc., Changzhou, Jiangsu, China)	Polylactic acid	~5 h (1 h pre-processing, 3–4 h printing)	NR	NR
Copeland et al. [30]	Ultimaker 2 Extended	Polylactic acid (PLA)	2 days (construction) 8–10 h (printing)	NR	NR
El Khoury et al. [31]	NR	Polyolefin materials (mixture of additive and polypropylene)	15 h	NR	NR
Exner et al. [32]	EOS Formiga	Polyamid	8 h (preoperative planning and guide design) 3 workdays to receive the printed molds	USD 2500 (planning and guide design) USD 600 (manufacturing)	NR
Eyiis et al. [33]	NR	NR	NR	EUR 590	NR
Grincuk et al. [34]	Zortrax M200Plus	Polylactic Acid (PLA)	NR	NR	NR
Guebeli et al. [35]	Atum 3D DLP Station	Photopolymer resin	145 min (scanning, modeling, printing, post-processing, and application)	USD 20/EUR 17.50	USD 30,000
Honigmann et al. [36]	Stratasys (Objet Eden 250)	Biocompatible UV curable acrylate (Med-610, Stratasys)	NR	NR	NR
Houdek et al. [37]	Polyjet 3D printer	Proprietary polymer	NR	NR	NR
Huang et al. [38]	UP Box 3D printer, Go Hot Technologies Co., Ltd., Taiwan	Polylactic Acid (PLA)	7 h	USD 4/model	NR
Inge et al. [19]	Ultimaker 3	NR	1 h	EUR 10/saw guide	EUR 3626
Jew et al. [39]	Dimension Elite 3-dimensional printer (Stratasys)	Acrylonitrile butadiene styrene	NR	NR	NR
Joo et al. [40]	FlashForge Creator Pro	Polylactic Acid (PLA)	< 2 h	USD 30	NR
Kim et al. [41]	FINEBOT Z420	Thermoplastic polyurethane	6 h	USD 70 USD/splint	NR
Kohlhauser et al. [42]	NR	NR	NR	NR	NR
Kong et al. [43]	NR	NR	NR	NR	NR
Krishna et al. [44]	Ultimaker S5 Bundle	Polylactic Acid (PLA)	NR	NR	NR
Kunz et al. [45]	Stratasys, Dimension SST	Thermoplastic acrylonitrile butadiene styrene	NR	NR	NR
Kuptniratsaikul et al. [46]	Mlab 200R	Titanium (Ti-6AI-4V grade)	NR	NR	NR
Lee et al. [47]	Fused filament fabrication type 3D printer	Acrylonitrile butadiene styrene (ABS) and thermoplastic polyurethane resin	1 day (measurement to prosthesis production)	USD 30	NR
Ma et al. [48]	NR	Photosensitive resin for model and titanium alloy (Ti-6Al-4V) for prosthesis	NR	NR	NR
Marcano-Fernandez et al. [49]	FORMIGA P 110 Velocis (EOS GmbH- Electro Optical Systems, Munich, Germany)	Polyamide, PA2200	6–8 h	EUR 330/guide	NR
Matter-Parrat and Liverneaux [5]	NR	NR	NR	NR	NR
Oka et al. [50]	Formiga P, EOS GmbH Electro Systems, Krailling, Germany or Eden250, Objet Geometries, Rehovot, Israel	Medical-grade resin	NR	USD 1500–2000 USD/model	NR
Oki et al. [51]	Uprint SE Plus	NR	NR	NR	NR
Osagie et al. [52]	NR	Polyethylene	144 h	GBP ~34/model	NA
Peeters et al. [53]	NR	NR	NR	NR	NR
Raeker-Jordan et al. [54]	Raise3D Pro2 printer series, FormLabs Form3 resin printer	Holden’s HX-80 latex, FormLabs resin	> 60 h (printing: 20 h, preparation: 10–15 h, curing and cooling: 30 h)	USD 80	NR
Roner et al. [55]	NR	NR	NR	NR	NR
Rossello [56]	NR	Titanium	NR	NR	NR
Samaila et al. [57]	3D printer (HP Design Jet 3D, Hewlett-Packard, Palo Alto, CA, USA)	Acrylonitrile butadiene styrene	4 h	EUR 50	NR
Schmidt et al. [58]	Formlabs (form 2 printer)	Clear photopolymer resin	NR	NR	NR
Schmidt et al. [59]	Formlabs 2 printer	Clear photopolymer resin	NR	NR	NR
Schutz et al. [60]	Fusion3 F410	Acrylonitrile butadiene styrene (ABS)	1.5 h (design) 25 min (printing)	USD 0.50/splint	NR
Schweizer et al. [61]	Materialise and Medacta (Castel San Pietro, Switzerland)	Polyamide (PA-12)	2–4 h	USD 220–320/case	NR
Sedigh et al. [62]	NR	Polylactic Acid (PLA)	NR	USD 300–900	NR
Shintani et al. [63]	Z Printer 650^®^; 3D Systems Inc., Valencia, CA, USA	NR	NR	NR	NR
Stefanovicet al. [64]	MakerBot Replicator1 Fused filament fabrication (FFF) 3D printer, Bq Witbox FFF 3D printer,	Polylactic Acid (PLA), Arnitel Eco	NR	NR	NR
Temmesfeld et al. [65]	M200 benchtop 3D printer (Zortrax)	1.75 mm proprietary ultra-filament	NR	USD 30–40/model	NR
Vijayan et al. [66]	Digital Light Processing 3D Printer	Photopolymer resin	NR	NR	NR
Wan et al. [67]	NR	MED610 biocompatible resin	12.5 h	USD ~420/design	NR
Wang et al. [68]	Electron beam melting technology (ARCAM Q10, Mölndal, Sweden)	Ultrahigh-molecular-weight polyethylene	2–4 w	NR	NR
Xiao et al. [69]	BY-3D-I instant 3D external fixation printer	Polyester fiber polymer material	25 min	NR	NR
Xie et al. [70]	NR	NR	NR	NR	NR
Xu et al. [71]	Zortrax M200	NR	NR	NR	NR
Yin et al. [72]	Stratasys Objet30 prime	Photopolymer of medical compatibility	30 h	USD 300/3D guiding plate	NR
Yuan et al. [73]	NR	Polyether ether ketone	NR	NR	NR

h: hours; m: months; NA: Not applicable; NR: Not reported; w: weeks, min: minutes.

## Data Availability

The original contributions presented in this study are included in the article and Supplementary Materials. Further inquiries can be directed to the corresponding author.

## Supplementary Materials

The following supporting information can be downloaded at: https://www.mdpi.com/article/10.3390/jpm15120611/s1, Table S1: Detailed Patient Characteristics; Table S2: PRISMA checklist.
