# Supplementary material for: Three-Dimensional Printing in Hand Surgery: What Is New? A Systematic Review
Source: J Pers Med. 2025 Dec 8;15(12):611. doi: 10.3390/jpm15120611 (PMC12734102; doi:10.3390/jpm15120611)
Supplement: Supplementary file 1 [file jpm-15-00611-s001.zip › SUPP DATA 1.pdf]

Supplementary Table S1. Detailed Patient Characteristics

Study	**N main group subjects**	**Controls**	**Anatomic Area of Interest**	**Total Patients**	**Mean Age (range)**	**Sex**		**Dominant Hand**	**Diagnosis**	**Time from injury**
						**M**	**F**			
Anderson et al. [23]	1	0	Entire hand	1	9	0	1	NR	Congenital hand deficiency	NA
Belloti et al. [24]	9	NR	Distal radius	9	47.5 (18-65)	5	4	3	Acute distal radius fracture	NR
Belloti et al. [25]	2	NR	Distal radius	2	46.5	1	1	NR	Distal radius malunion/non-union	2.2 yr
Bizzotto et al. [13]	40	NR	Distal Radius	40	NR	NR		NR	Acute distal radius fracture	NR
Bizzotto et al. [3]	31	NR	Distal Radius	102	NR (20-78)	45	57	NR	Distal radius fracture	NR
Brichacek et al. [26]	10	NR	Metacarpals and phalanges	10	NR	NR	NR	NR	Bennett's fracture, fifth metacarpal neck and proximal phalanx fracture	NR
Casari et al. [27]	7	Contra-lateral healthy hand	Distal radius	7	47.7	5	2	4	Acute distal radius fracture	3-4 days
Chen et al. [11]	10	NR	Distal radius, ulnar styloid	10	NR (5-78)	4	6	NR	Acute distal radius fracture	NR
Chen et al. [28]	55	52	Distal radius	107	27.69	30	22	NR	Acute distal radius fracture	3-4 days
Chen et al. [29]	23	25	Distal radius	48	38.78	14	9	NR	Acute distal radius fracture	3-4 days
Chen et al. [7]	1	0	Distal radius	1	58	1	0	NR	Osteosarcoma	6 m
Copeland et al. [30]	1	0	Entire hand	1	59	1	0	1	Transradial amputation	12 m

El Khoury et al. [31]	18	16	Distal radius	34	56	13	21	17	Acute distal radius fracture	2 w
Exner et al. [32]	1	0	Distal radius	1	39	0	1	NR	Clear cell sarcoma	6 years
Eyiis et al. [33]	24	25	Trapezoid-metacarpal joint	49	63	12	37	24	Trapeziometacarpal osteoarthritis	NR
Grincuk et al. [34]	33	33	Distal radius	66	50.6 (18-75)	27	39	33	Acuate distal radius fracture	< 3 w
Guebeli et al. [35]	19	20	Distal radius	39	49 (18-78)	23	16	20	Acute distal radius fracture	3-5 days
Honigmann et al. [36]	1	NR	Distal radius	1	54	1	0	1	Distal radius malunion/non-union	NR
Houdek et al. [37]	1	NR	Scaphoid	1	26	1	0	NR	Scaphoid malunion/non-union	9 m
Huang et al. [38]	1	0	Distal radius	2	35 (23-46)	1	0	NR	Acute distal radius fracture, FPL rupture	NR
Inge et al. [19]	1	NR	Distal Radius	1	16	0	1	NR	Distal radius malunion/non-union	3 m
Jew et al. [39]	4	NR	Scaphoid	4	31	4	0	NR	Acute scaphoid fracture, scaphoid malunion/non-union	5.6 m
Joo et al. [40]	1	0	Palmer hand and forearm	1	45	1	0	NR	4th degree burn	560 days
Kim et al. [41]	11	11	Wrist	22	33.8 (19–65)	2	20	NR	Degenerative wrist	NR
Kohlhauser et al. [42]	1	0	5th metacarpal	1	57	1	0	NR	Extensive osseous defect of 5th metacarpal bone	NR
Kong et al. [43]	16	16	Distal radius	32	41.1	10	6	7	Acute distal radius fracture	NR

Krishnal et al. [44]	1	0	Distal radius	1	18	1	0	NR	Osteoid osteoma	3 yr
Kunz et al. [45]	9	NR	Distal radius	9	NA	NR		NR	Acute distal radius fracture	8 m
Kuptniratsaikul et al. [46]	1	NR	Distal radius	1	34	0	1	1	Tumor	6 m
Lee et al. [47]	2	0	Phalanges	2	25.5 (25-26)	1	1	NR	Proximal interphalangeal joint amputation	2 m
Ma et al. [48]	5	NR	Lunate	5	51.6 (37-64)	2	3	NR	Kienböck	11.4 (1-24) m
Marcano-Fernandez et al. [49]	10	10	Scaphoid	20	25	10	0	NR	Acute scaphoid fracture	NR
Matter-Parrat & Liverneaux [5]	1	NR	Distal Radius	1	16	1	0	0	Distal radius malunion/non-union	36 m
Oka et al. [50]	5	NR	Distal radius	5	44.4 (33-76)	5	0	NR	Distal radius malunion/non-union	9 (6-12) m
Oki et al. [51]	1	0	Scaphoid	1	34	1	0	NR	Scaphoid fracture non-union	1.5 yr
Osagie et al. [52]	3	NR	Scaphoid, distal radius, carpus	3	34.3	3	0	3	Scaphoid malunion/non-union (N=1), ulnar malunion/non-union (N=1), degenerative wrist (N=1)	2 yr
Peeters et al. [53]	1	NR	Scaphoid	1	30	1	0	NR	Scaphoid malunion/non-union	14 m
Raeker-Jordan et al. [54]	NA	NA	Distal radius	0	NA	NA	NA	NA	Distal radius fracture	NA
Roner et al. [55]	8	7	Distal radius	15	42 (30-66)	NR		NR	Distal radius malunion/non-union	5.8 yr

Rossello [56]	1	0	Scaphoid	1	18	1	0	NR	Scaphoid non-union	2 yr
Samaila et al. [57]	10	NR	Distal radius	52	NR	NR		NR	Acute distal radius fracture	NR
Schmidt et al. [58]	2	NR	Scaphoid	2	28.5	2	0	NR	Scaphoid avascular necrosis	NR
Schmidt et al. [59]	1	0	Metacarpal	1	47	0	1	NR	Giant cell tumor (GCT)	3.5 yr
Schutz et al. [60]	2	NR	Wrist, metacarpals, phalanges	2	36 wk gestational age - term	2	0	NR	Skeletal dysplasia, chromosomal abnormalities	NA
Schweizer et al. [61]	6	NR	Distal radius	6	48	4	2	4	Acute distal radius fracture intra-articular	9 m
Sedigh et al. [62]	2	0	Phalange, metacarpal	2	27 (20-34)	2	0	1	Phalange, metacarpal fractures	10-14 days
Shintani et al. [63]	19	NR	Distal radius	19	55.1 (29.3-81.3)	6	13	NR	Distal radius malunion/non-union	1.5 yr
Stefanovicet al. [64]	1	0	1st metacarpal	1	40	1	0	0	Congenital absence of the thumb	NA
Temmesfeld et al. [65]	1	NR	Distal radius	1	18	1	0	0	Distal radius malunion/non-union	12 m
Vijayan et al. [66]	1	0	Phalanges	1	65	1	0	NR	Proximal interphalangeal joint amputation	2 yr
Wan et al. [67]	10	NR	Scaphoid	4	NA	4	0	NR	Acute scaphoid fracture	NR
Wang et al. [68]	15	15	Distal radius	30	38	6	9	NR	Tumor	NR
Xiao et al. [69]	20	20	Distal radius	40	45	16	24	NR	Colles fracture	12 h

Xie et al. [70]	1	NR	Lunate	1	41	NR		NR	Kienböck	2 yr
Xu et al. [71]	21	NR	Distal radius	21	38.8	19	2	NR	Acute distal radius fracture	NR
Yin et al. [72]	8	8	Scaphoid	16	28 (20-60)	8	0	NR	Scaphoid malunion/non-union	5.2 yr
Yuan et al. [73]	1	0	Lunate	1	42	0	1	NR	Kienböck	5 m

NR: not reported, NA: not applicable, N: number; M: male, F: female, m: months, yr: year, h: hour, w:week, FPL: flexor pollicis longus
